# Supplementary material for: A toolbox of nanobodies developed and validated for use as intrabodies and nanoscale immunolabels in mammalian brain neurons
Source: eLife. 2019 Sep 30;8:e48750. doi: 10.7554/eLife.48750 (PMC6785268; doi:10.7554/eLife.48750)
Supplement: Supplementary file 1. — Table lists the screening results from prior monoclonal antibody (mAb) projects and the results from the corresponding nanobody screens. The numbers in parentheses in the final three columns of the nanobody rows represent the number of expected application-positive nanobodies obtained from the population of unique ELISA-positive nanobodies tested, based on percentages in mouse mAb projects. [file elife-48750-supp1.docx]

**Supplementary Table 1. Summary of corresponding mouse monoclonal antibody and nanobody immunolabeling results**

| **Target** | **Project** | **ELISA positives** | **COS-1 ICC positives** | **Brain IHC positives** | **Brain IB positives** |
| --- | --- | --- | --- | --- | --- |
| Homer1 | mAb L113 | 144 | 144 (100%) | 73 (51%) | 29 (20%) |
|  | nAb | 39 | 33 (39) | 25 (20) | 13 (8) |
| IRSp53 | mAb L117 | 144 | 115 (80%) | 85 (59%) | 37 (26%) |
|  | nAb | 17 | 0 (14) | 0 (10) | 0 (4) |
| SAPAP2 | mAb N459 | 96 | 80 (83%) | 14 (15%) | 19 (20%) |
|  | nAb | 15 | 0 (12) | 4 (2) | 0 (3) |
| Gephyrin | mAb L106 | 96 | 56 (58%) | 37 (39%) | 34 (35%) |
|  | nAb | 24 | 0 (9) | 1 (9) | 2 (8) |
| AMIGO-1 | mAb L86, L86A | 122 | 21 (17%) | 33 (27%) | 17 (14%) |
|  | nAb | 18 | 0 (3) | 0 (5) | 0 (2) |
