# Supplementary material for: A toolbox of nanobodies developed and validated for use as intrabodies and nanoscale immunolabels in mammalian brain neurons
Source: eLife. 2019 Sep 30;8:e48750. doi: 10.7554/eLife.48750 (PMC6785268; doi:10.7554/eLife.48750)
Supplement: Supplementary file 2. — Table lists nanobody project target, fragment used for llama immunization and tag. [file elife-48750-supp2.docx]

**Supplementary Table 2. Immunogens used for llama immunization**

| **Immunogen** | **Fragment region (UniProt ID)** | **Tag** |
| --- | --- | --- |
| Homer1 | amino acids 121-363 (C-terminus) of mouse Homer1L (Q9Z2Y3) | - |
| IRSp53 | amino acids 1-250 (N-terminus) of human IRSp53/BAIAP2 (Q9UQB8) | GST |
| SAPAP2 | amino acids 76-244 (N-terminus) of rat SAPAP2 (P97837) | - |
| Gephyrin | amino acids 1-181 (N-terminus) of human Gephyrin (Q9NQX3) | - |
| AMIGO-1 | amino acids 395-493 (cytoplasmic C-terminus) of mouse AMIGO-1 (Q80ZD8) | GST |
