# Supplementary material for: A toolbox of nanobodies developed and validated for use as intrabodies and nanoscale immunolabels in mammalian brain neurons
Source: eLife. 2019 Sep 30;8:e48750. doi: 10.7554/eLife.48750 (PMC6785268; doi:10.7554/eLife.48750)
Supplement: Supplementary file 3. — Table lists primers used to amplify llama heavy chain variable regions. [file elife-48750-supp3.docx]

**Supplementary Table 3. Primers used for heavy chain repertoire cloning**

| **Forward primers** |  |
| --- | --- |
| VH1 (Sfi I) | 5’-CATGCCATGACTCGCGGCCCAGGCGGCCATGGCCCAGGTGCAGCTGGTGCAGTCTGG-3’ |
| VH3 (Sfi I) | 5’-CATGCCATGACTCGCGGCCCAGGCGGCCATGGCCGAGGTGCAGCTGGTGGAGTCTGG-3’ |
| VH4 (Sfi I) | 5’-CATGCCATGACTCGCGGCCCAGGCGGCCATGGCCCAGGTGCAGCTGCAGGAGTCGGG-3’ |
| F1 (Sfi I) | 5’-CATGCCATGACTGTGGCCCAGGCGGCCATGCAGKTGCAGCTCGTGGAGTC-3’ |
| F2 (Sfi I) | 5’-CATGACTGTGGCCCAGGCGGCCATGCAGGTGCAGCTCGTGGASWCHGGNGGAGGMTTGGT-3’ |
| VHH1BACK (Sfi I) | 5’-GTTATTACTCGCGGCCCAGGCGGCCATGGCCCAGGTSMARCTGCAGSAGTCWGG-3’ |
| CHH6BACK (Sfi I) | 5’-T GTTATTATCTGCGGCCCAGGCGGCCATGGCCGATGTGCAGCTGCAGGCGTCTGGRGGAGG-3’ |
| **Reverse primer** |  |
| JH | 5’-CCACGATTCTGGCCGGCCTGGCCTGAGGAGACRGTGACCTGGGTCC-3’ |
