# Supplementary material for: A toolbox of nanobodies developed and validated for use as intrabodies and nanoscale immunolabels in mammalian brain neurons
Source: eLife. 2019 Sep 30;8:e48750. doi: 10.7554/eLife.48750 (PMC6785268; doi:10.7554/eLife.48750)
Supplement: Supplementary file 4. — Table lists non-nanobody antibodies used throughout this study, the immunogen used in their development, the species and IgG subclass, the manufacturer and Antibody Registry/RRID information, and the figure in which each antibody was used. [file elife-48750-supp4.docx]

**Supplementary Table 4. Non-nanobody antibodies used in this study**

| **Antibody name and target** | **Immunogen** | **Species/isotype** | **Manufacturer information (RRID)** | **Figure** |
| --- | --- | --- | --- | --- |
| L113/130 Homer1L | amino acids 121-363 (C-terminus) of mouse Homer1L | Mouse/IgG1 | NeuroMab catalog # 73-454 (RRID:AB_2629419) | 1-S1,1-S2,4,6 |
| L117/1  IRSp53 | amino acids 1-250 (N-terminus) of human IRSp53 | Mouse/IgG1 | NeuroMab catalog # 73-449 (RRID:AB_ 2619741) | 1-S2 |
| N459/94  SAPAP2 | amino acids 76-244 (N-terminus) of rat SAPAP2 | Mouse/IgG1 | Trimmer lab in house (RRID:AB_2797391) | 1-S2 |
| L106/23  Gephyrin | amino acids 1-181 (N-terminus) of human Gephyrin | Mouse/IgG2b | Trimmer lab in house (RRID:AB_2783821) | 1-S3 |
| L86A/37  AMIGO-1 | amino acids 395-493 (cytoplasmic C-terminus) of  mouse AMIGO-1 | Mouse/IgG2a | NeuroMab catalog # 73-329 (RRID:AB_2315801) | 1-S3 |
| K28/43  PSD-95 | amino acids 77-299 (PDZ domains 1 and 2) of human PSD-95 | Mouse/IgG2a | NeuroMab catalog # 73-028 (RRID:AB_ 10698024) | 1-S4 |
| L125/129  Pan-Synapsin | amino acids 76-417 (actin-binding and synaptic-vesicle binding domains) of human Synapsin III | Mouse/IgG2b | NeuroMab catalog # 73-457 (RRID:AB_2728740) | 1-S5 |
| K89/34  Kv2.1 | amino acids 837-853 (HMLPGGGAHGSTRDQSI, cytoplasmic C terminus) of rat Kv2.1 | Mouse/IgG1 | NeuroMab catalog # 73-014  (RRID:AB_10672253) | 1-S5 |
| Anti-Homer1 | amino acids 1-196 (N-terminus) of human Homer1 | Rabbit pAb | Synaptic Systems catalog # 160003 (RRID:AB_887730) | 2 |
| Anti-GFP | recombinant GFP | Chicken pAb | Aves catalog # GFP-1020 (RRID:AB_10000240) | 2 |
| AP-20  MAP2 | Purified bovine MAP2 | Mouse/IgG1 | Sigma catalog # M1406 (RRID:AB_477171) | 2 |
| 16B12  HA (Alexa 488) | HA tag | Mouse/IgG1 | ThermoFisher Cat# A-21287 (RRID:AB_2535829) | 1-S6,4 |
| L113/27  Homer1L | amino acids 121-363 (C-terminus) of mouse Homer1L | Mouse/IgG2b | NeuroMab catalog # 73-453 (RRID:AB_2629418) | 1-S6,4,5 |
| MAP2 | three KLH-conjugated peptides from N-and C-terminal regions of rat MAP-2 | Rabbit pAb | Millipore-Sigma Cat# AB5662-I (RRID:AB_2800501 | 5 |
| 2-2.2.14  HA (Alexa 647) | HA tag | Mouse/IgG1 | ThermoFisher Cat# 26183-A647 (RRID:AB_2610626) | 5 |
| L106/93 Gephyrin | amino acids 1-181 (N-terminus) of human Gephyrin | Mouse/IgG2b | NeuroMab catalog # 73-444 (RRID:AB_2617121 | 6 |
| 3F10  HA (HRP) | HA tag | Rat/IgG1 | Sigma-Aldrich Cat# 12013819001 (RRID:AB_390917) | 6 |
| 12CA5  HA | HA tag | Mouse/IgG2b | Trimmer lab in house (RRID:AB_2532070) | NA |
